# Effects of human immunoglobulin A on *Cryptococcus neoformans* morphology and gene expression

**DOI:** 10.1128/spectrum.02008-24

**Published:** 2025-02-21

**Authors:** Nuria Trevijano-Contador, Haroldo C. de Oliveira, Claudia Malacatus-Bravo, Varona Sarai, Isabel Cuesta, Marcio L. Rodrigues, Óscar Zaragoza, Liise-anne Pirofski

**Affiliations:** 1 Mycology Reference Laboratory, National Centre for Microbiology, Instituto de Salud Carlos III, Carretera Majadahonda-Pozuelo, Madrid, Spain; 2 Instituto Carlos Chagas, Fundação Oswaldo Cruz, Curitiba, Brazil; 3 Bioinformatics Unit, Core Scientific and Technical Units, Instituto de Salud Carlos III, Madrid, Spain; 4 Instituto de Microbiologia Paulo de Góes, Universidade Federal do Rio de Janeiro, Rio de Janeiro, Brazil; 5 Center for Biomedical Research in Network in Infectious Diseases (CIBERINFEC-CB21/13/00105), Instituto de Salud Carlos III 38176 , Madrid, Spain; 6 Division of Infectious Diseases, Department of Medicine, Albert Einstein College of Medicine, Bronx, New York, USA; Virginia-Maryland College of Veterinary Medicine, Blacksburg, Virginia, USA

**Keywords:** *Cryptococcus neoformans*, human immunoglobulin, IgA, IgG, IgM, Titan-like cells, natural antibodies, cryptococcosis, extracellular vesicles, RNAseq, gene expression, extracellular vesicles

## Abstract

Human IgM was previously shown to inhibit Titan-like cell formation of *Cryptococcus neoformans*, whereas IgG did not. Here, we conducted an in-depth analysis of the effect of normal human IgA on *C. neoformans* biology (strain H99) and compared it to that of IgG and IgM. We found that like IgM, IgA affected H99 cell size and morphology. The total size of cells cultured with IgA was significantly smaller at 24 h than cells cultured with IgM and IgG and comparable to IgM but smaller than IgG at 72 h. We also examined extracellular vesicle (EV) production and found that it was significantly reduced in cells cultured with IgA than the control, but the EVs were larger. To further probe the effect of IgA on H99, we performed expression profiling by RNAseq of H99 cells cultured with each immunoglobulin isotype and compared the results with IgA to those with IgM, IgG, and a control (PBS). These comparisons showed that cells cultured with IgA overexpressed genes related to cell rescue, defense, virulence, energy conservation, adapation to stress with CNAG_00735 (aldehyde dehydrogenase family seven member A1) being the most overexpressed, and repressed some genes related to vesicular transport, including CNAG_04306 (vesicle transporter SFT2B) and CNG_00063 (histone H3). Collectively, our findings suggest that the effects of IgA on cryptococcal biology deserve further investigation, as they reveal new insights into human host–*C. neoformans* interaction, which suggest that antibody responses may affect gene expression in *C. neoformans*.

**IMPORTANCE:**

Profound CD4 T cell deficiency is associated with the development of cryptococcosis in HIV-infected individuals. However, perturbations in antibody immunity, including reduced levels of plasma IgA and IgM, have also been associated with cryptococcal disease status. While IgM has been studied in some detail, IgA has not. Here, we evaluated the effect of normal human IgA on *Cryptococcus neoformans* biology and morphology to expand knowledge of the role that it may play in cryptococcal pathogenesis.

## INTRODUCTION

Invasive fungal infections, which are on the rise globally (1–3), cause an estimated 6.5 million cases and 3.8 million deaths annually (4) and are associated with mortality between 25-70% and high healthcare costs (5). *Cryptococcus neoformans*, first on the 2022 World Health Organization list of critical priority fungal pathogens (6), causes devastating meningoencephalitis in immunocompromised individuals, especially those with HIV infection, as well as organ transplant recipients and those on biologics (7–9). Yeasts of the *Cryptococcus* genus are environmental microbes that are acquired by humans early in life after which they assume a state of latency (10–12). The development of cryptococcosis generally reflects escape from latency (10). Cryptococcosis, particularly cryptococcal meningoencephalitis, is associated with high mortality, unless it is diagnosed and treated early. In 2024, it was estimated that there were 194,000 cases of cryptococcal meningitis and 147,000 deaths (75.8%) globally, primarily in HIV-infected persons in Sub Saharan Africa (4).

*C. neoformans* has numerous virulence attributes, including its key virulence factor, a polysaccharide capsule composed mainly of glucuronoxylomannan (GXM) and a complex network of polysaccharides that surround the cell wall (13–15). The capsule enhances disease by shielding the fungal cell from attack from the host immune system and shed, and glucuronoxylomannan inhibits host immune responses. In addition, *C. neoformans* can undergo a morphological change that is manifested by an increase in both cell and capsule size, resulting in enlarged cells, called Titan cells (16, 17), which can reach a size of 80–100 μm *in vivo* (16–19). *In vitro* conditions for inducing “Titan-like cells” result in cells of 30–50 μm (20–22).

The human immunoglobulin isotypes, IgM, IgG, IgA, IgE, and IgD, differ based on their heavy-chain constant regions (23). IgM is a pentamer. Serum IgA is largely monomeric and mucosal (secretory) IgA is largely dimeric. The other immunoglobulins are monomeric. Mucosal IgA is an important first line of defense and serum IgA can mediate anti-microbial activity (24, 25). Prior work has shown that plasma levels of IgA and laminarin (LAM, a mainly 1–3 D beta glucan)-IgA were lower in HIV-infected individuals that had cryptococcal meningitis than those who did not and inversely associated with cryptococcal meningitis status in one study (26, 27). These findings led us to investigate the effect of human IgA on cryptococcal biology. A previous study showed that human IgM inhibited formation of Titan-like cryptococcal cells *in vitro* and induced changes in *C. neoformans* capsule size and cell wall morphology (28). In this study, we compared the effects of IgA, IgM, and IgG on *C. neoformans* morphology, gene expression, and extracellular vesicle production, with a focus on the effects of IgA. Our results show that human IgA can induce morphological and biological changes in *C. neoformans* cells cultured *in vitro*.

## MATERIALS AND METHODS

### *Cryptococcus neoformans* strains and media

*Cryptococcus neoformans* strain H99 (29) was used. H99 cells were grown in Sabouraud medium at 30°C with shaking at 150 rpm overnight. The next day, the cells were washed twice with phosphate-buffered saline (PBS) and transferred back to Sabouraud liquid medium at a final concentration of 10^6^ cell/mL with 50 µg/mL of human IgM, IgG, or IgA (Sigma, USA) and grown at 30 or 37°C with different incubation times depending on the experiment.

### Human immunoglobulins

Human serum IgM, IgG, and IgA were obtained from a commercial source (Sigma, USA). Stock solutions were prepared at a concentration of 1 mg/mL and dialyzed to remove sodium azide using a Slide-A-Lyzer MINI Dialysis Devices (Thermo Scientific). A final concentration of 50 µg/mL was used in co-culture experiments with H99. This concentration was chosen because it was used in a prior study, which demonstrated that human IgM could inhibit *C. neoformans* (H99) Titan-like cell formation *in vitro* (28).

### Growth of H99 with IgM, IgG, and IgA

H99 cells were harvested by centrifugation and washed twice with PBS. Cellular suspensions were prepared at 2 × 10^5^ cells/mL in Sabouraud liquid containing 50 µg/mL of dialyzed IgM, IgG, and IgA. As a control, cell growth was monitored in Sabouraud and Sabouraud + dialyzed azide. Cells were cultured in triplicate in 96-well plates. Optical density was measured at 540 nm every hour at 30°C for 72 h with shaking for 5 s before each measurement. This experiment was performed three times on three different days.

### Titan-like cell induction assay

To induce Titan-like cells, H99 was grown in Titan cell medium (TCM) as previously described (20). Briefly, after growth in liquid Sabouraud medium overnight, cells were transferred to TCM (5% Sabouraud and 5% inactivated fetal bovine serum buffered with 50 mM MOPS with 15 µM sodium azide, Sigma, USA) to a final concentration of 5 × 10^4^ cells/mL at 37°C and 5% CO_2_ for 18 h. This experiment was performed three times on three different days.

### Visualization and measurement of H99 size

To measure H99 size, 10 µL of cells was mixed with an India ink drop (India ink reagent droppers; BD) and viewed under a Leica DMI 3000B microscope as previously described (30). Total cell size was defined as the size of the cell body plus the size of the capsule. At least five different fields were photographed with a Leica DFC 7000 camera and processed with Adobe Photoshop 7.0 (San Jose, CA).

### Extracellular vesicle (EV) isolation from solid medium

EVs were isolated from solid medium as previously described (31). This method has been shown to produce results comparable to EV isolation from liquid medium in less time (32). Briefly, one colony of H99 cultivated on solid Sabouraud medium was inoculated in Sabouraud liquid medium for 24 h at 30°C. Then, the cells were counted and adjusted to 1 × 10^7^ cells/plate on Sabouraud agar supplemented with 50 µg/mL of IgM, IgG, or IgA lyophilized and resuspended in PBS (three plates for each) and a control without antibody. The plates were incubated for 15 h at 37°C after which EVs were isolated following the protocol described in (31). For each condition, the cells were gently recovered from each of the three plates with an inoculation loop and suspended in 30  mL of PBS. The suspensions were centrifuged at 5,000×*g* for 15  min at 4°C for removal of the cells. To remove debris, the supernatants were collected and centrifuged again at 15,000×*g* for 15  min at 4°C. The resulting supernatants were filtered through 0.45 µm pore syringe filters and centrifuged at 100,000×*g* for 1  h at 4°C for pelleting down the EVs. EVs were resuspended in 100 µL of PBS and stored at 4°C before the Nanoparticle tracking analysis (NTA). This experiment was performed three times on three different days with three triplicates per experiment. The number of EVs produced per cell was analyzed based on the concentration of cells in 30 mL of PBS after their recovery from the Sabouraud plates together with the particle concentration after analyzing the samples by NTA.

### Nanoparticle tracking analysis of EVs

Nanoparticle tracking analysis was used to evaluate EV dimensions and concentration. This analysis was performed on an LM10 nanoparticle analysis system coupled with a 488 nm laser and equipped with an SCMOS camera and a syringe pump (Malvern Panalytical, Malvern, United Kingdom). The data were acquired and analyzed using the NTA 3.0 software (Malvern Panalytical). NTA histograms were also used for the quantification of the distribution of EVs sizes using the ImageJ software (https://imagej.nih.gov/ij/).

### RNA extraction for RNA-seq and sequencing

H99 was grown in 10 mL of liquid Sabouraud medium with a cell concentration of 10^6^ cells/mL and 50 µg/mL of human IgM, IgG or IgA (Sigma, USA) at 30°C with shaking 150 rpm during 6 h. The RNA extraction was performed using the Trizol reagent protocol (Ambion RNA, Life Technologies) with some modifications. Trizol was added to the samples and maintained on ice. Cells were bead-beaten for 3 min with FastPrep-24 (MP), alternating 20 s beating with 1 min on ice. The RNAs were quantified using QuantiFluor RNA System, and the quality was measured on Agilent 2100 Bioanalyzer. The kit used for RNA treatment and library preparation was IIIumina Stranded mRNA Prep (Illumina). An Illumina NextSeq 500 was used for sequencing (20). Original FASTQ documents were deposited at NCBI (BioProject ID PRJNA1191853).

### Transcriptomic data analysis

The bioinformatic analysis of the samples was performed by the Bioinformatics Department of the Carlos III Health Institute. The FastQ files were analyzed with an in-house RNA-seq pipeline (https://github.com/BU-ISCIII/rnaseq-nf) written in Nextflow (https://www.nextflow.io/) and based on the nf-core (https://nf-co.re/) previously written RNA-seq pipeline (https://github.com/nf-core/rnaseq). FastQ files containing raw reads were first analyzed for quality using fastQC v0.11.8 (http://www.bioinformatics.babraham.ac.uk/projects/fastqc/). Then raw reads were trimmed for low-quality 3′ ends and adapter sequence removal using Trim-momatic v.0.38 (33). High-quality reads were then aligned against the *C. neoformans* H99 reference genome (GCA_000149245.3) using STAR v2.6.1d (34), and alignment quality control was performed using RseQC v3.0.0 (35). Finally, transcriptome prediction and gene quantification were calculated using Subread’s featureCounts package v1.6.4 (36).

Three biological replicates for cells grown with IgA, IgM, IgG, or PBS were performed and analyzed to identify differentially expressed genes using the DESeq2 R/Bioconductor package v1.18.1 (33). DESeq2 was also used for normalization and result visualization. Genes with an adjusted *P* value (FDR/Benjamini–Hochberg) < 0.05 were selected. The products of this test were recovered as Annotated Probe Report, which included the coding sequences (overlapping option), and processed in Excel format. To identify functional families significantly overrepresented among the differentially expressed genes, the lists of genes were submitted to Gene Ontology and FunCat analyses at the FungiFun server (34). To this end, unless otherwise stated, a Fisher’s exact test with the significance level set to 0.05 and the Benjamini–Hochberg adjustment method were used. Due to the large number of cryptococcal gene products lacking functional annotation in the databases, a Blastp search was conducted against the proteomes of several different, relatively well-annotated fungi to expand the available functional information on the relevant genes.

### Statistical analysis

Statistical analyses were performed with GraphPad Prism 9 (GraphPad Software, Inc., San Diego, CA). The normality of each simple was first assessed using the Kolmogorov–Smirnov test (nonnormal distribution when *P* value was <0.1). Differences were estimated using analyses of variance (ANOVAs) and Dunnett’s and Student’s *t* tests. For nonparametric distributions, the Kruskal–Wallis and Mann–Whitney tests were used. The differences were calculated using a two-way analysis of variance for gene expression.

## RESULTS

### Effect of human, IgA, IgM and IgG on cell growth

**Fig 1:**
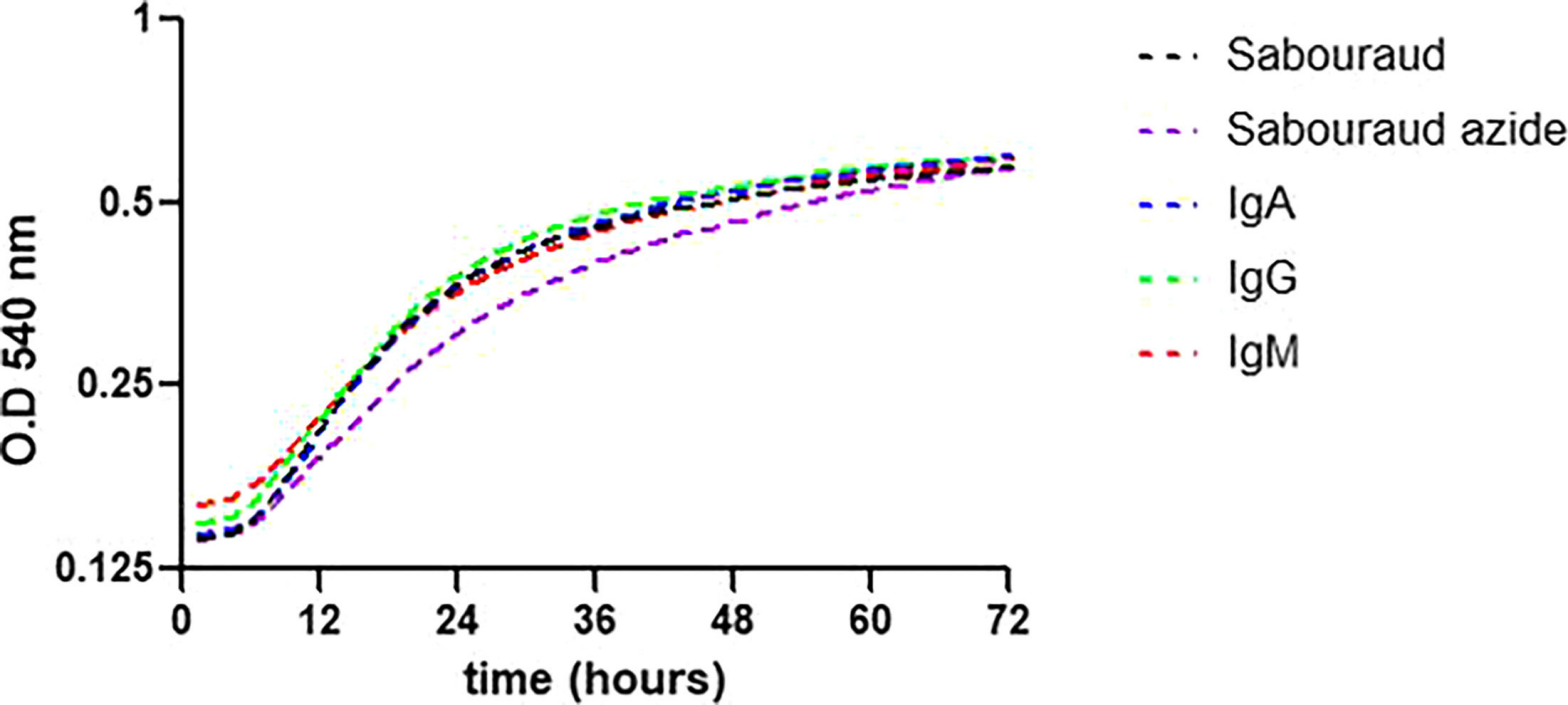
Growth curves in Sabouraud liquid medium at 30°C of *C. neoformans* H99 strain for 72 h. Growth of H99 cultured with a control and the immunoglobulins in the legend, depicted by OD_540_ on the Y axis, was determined at the times indicated on the Y axis. The experiments were performed three different times on three different days with three triplicates.

We performed growth curves of H99 cultured with human IgA, IgM, and IgG in Sabouraud liquid medium for 72 h at 30°C (Fig. 1). The growth of H99 was equivalent for the cells cultured with each immunoglobulin (IgM, IgG, and IgA) and comparable to that of controls, Sabouraud, and Sabouraud azide. The latter condition was included to ensure that the small amount of azide in Sabouraud medium did not affect cell growth.

### Human IgA affects H99 cell size

**Fig 2:**
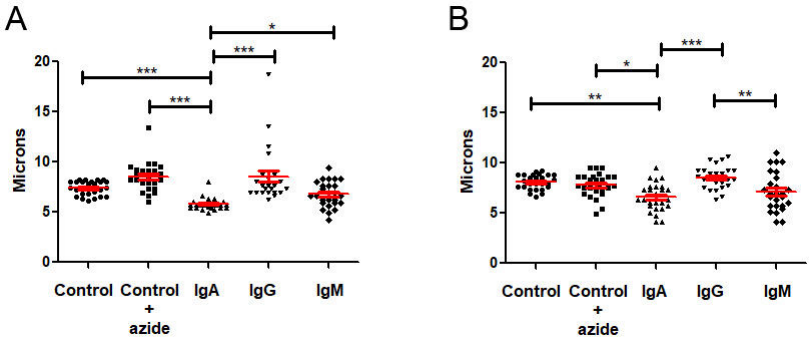
Effect of human immunoglobulins on the total cell size of H99. The total size of H99 is the sum of the cell body plus the capsule size. Y axis depicts the size in microns of *C. neoformans* H99 strain cells cultured in Sabouraud medium at 30°C for 24 (**A**) and 72 h (**B**) after culture with human IgA, IgG, and IgM and controls (Sabouraud and Sabouraud + azide). The red lines mark the mean and standard error. Asterisks indicate significant differences (*P* < 0.05), one-way ANOVA. The experiments were performed three different times on three different days with three replicates.

Light microscopic images of H99 cells cultured with IgM, IgG, and IgA were taken after 24 h of growth in Sabouraud medium to observe the morphology and measure the size of the cells. The total size of the cells defined as the size of the cell body plus the capsule cultured with the immunoglobulins was determined after 24 and 72 h of growth at 30°C in Sabouraud (Fig. 2A and B respectively). For cells cultured with IgA, the total size of the cells was significantly smaller, with a mean of five μm, compared to the controls (Sabouraud and Sabouraud + azide) and the cells cultured with IgG and IgM after 24 h (Fig. 2A). After 72 h, the sizes of the cells cultured with IgA and IgM were similar, while the cells cultured with IgG were larger (Fig. 2B).

### Human IgA inhibits H99 Titan-like cell formation *in vitro*

**Fig 3:**
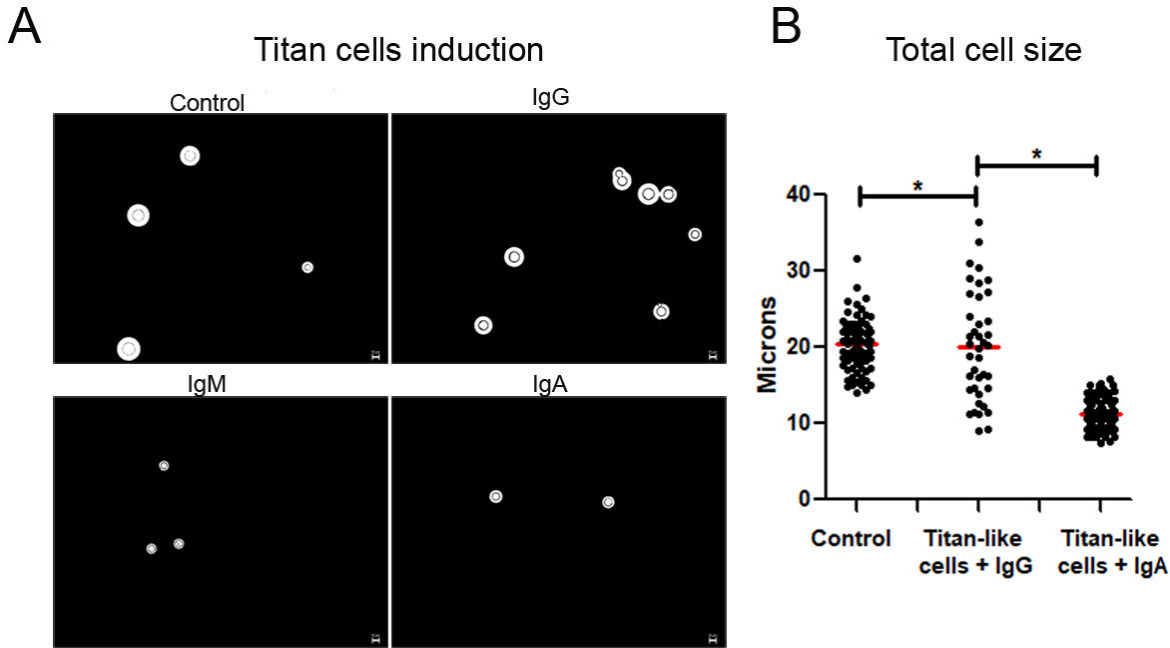
Cellular total sizes and size distribution of H99 grown in TCM medium. (**A**) Total cellular size of H99 in TCM with PBS (control) and IgG (upper panels) and IgM and IgA (lower panels). The total size of H99 is the sum of the cell body plus the capsule size. The images were obtained on a Leica microscope with the 40× objective. (**B**) Size distribution of the total cell body in TCM for Titan-like cell induction after culture of *C. neoformans* with human IgG and IgA for 18 h at 37°C with 5% of CO_2_. Asterisks indicate significant differences (*P < 0.05). One-way ANOVA and Dunnett’s multiple-comparisons test. The bars represent the standard errors of the means. Scale bar represents 10 µm. The experiments were performed three different times on three different days with three triplicates.

Culture of H99 with IgA resulted in smaller total cell sizes compared to cells incubated with IgG, IgM, and control (Fig. 3A). The frequencies of Titan-like cells larger than 20 µm were 60% for control, 40% for IgG, and <15% for cells cultured with IgA. The mean total cell size of cells grown in TCM with IgA was 10 μm, while cells grown with IgG or the control the sizes reached 20 μm (Fig. 3B). Therefore, IgA and IgM inhibited Titan-like cell formation, whereas IgG did not.

### EV production by H99 cultured with IgM, IgG, and IgA

**Fig 4:**
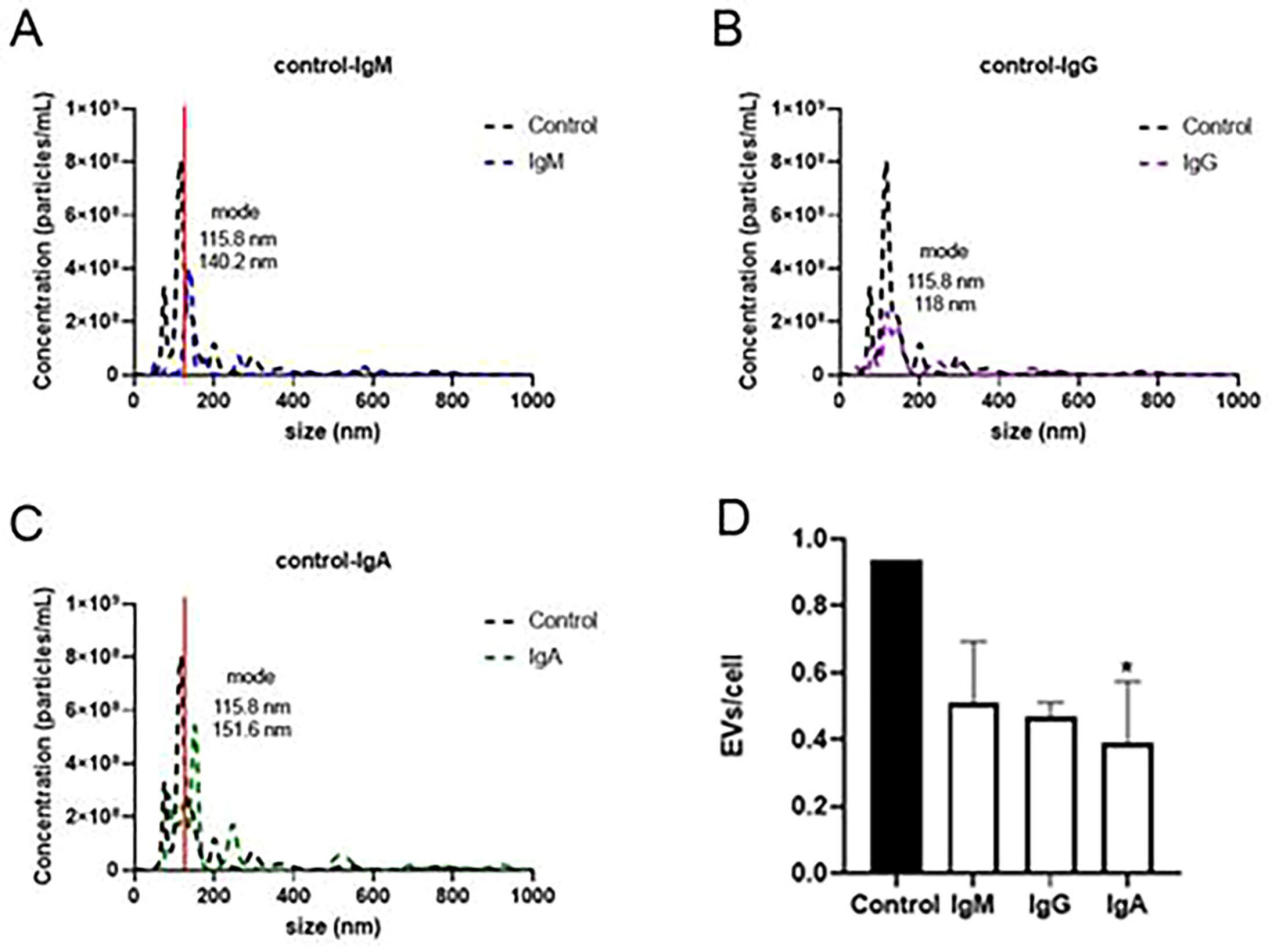
Nanoparticle tracking analysis of H99 cultured with human IgG, IgM, and IgA. Representative histograms of the average size distribution of (**A**) control–IgM, (**B**) control–IgG (**B**), and (**C**) control–IgA. (**D**) Quantification of EVs/cell production. (**D**) Number of EVs per cell (X axis) for each of the conditions on the Y axis (*P < 0.05 compared to control). One-way ANOVA and Dunnett’s multiple-comparisons test. The bars represent the standard errors of the means. The red line in panels A and C highlights the difference in the modes between IgM (panel A) and IgA (panel C). The experiments were performed three different times on three different days with three triplicates.

H99 cells cultured with IgM, IgG, and IgA produced fewer EVs than the control. For cells cultured with IgM, the concentration was 4 × 10^8^ particles/mL, with IgG ~2 × 10^8^ particles/mL and with IgA ~5 × 10^8^ particles/mL (Fig. 4A through C). The mode of the EV sizes of the cells cultured with IgG and the control was comparable at 118 and 115 nm, respectively. However, EVs recovered from cells cultured with IgM and IgA were larger than the control and cells cultured with IgG (118 nm); 140.2 nm with IgM and 151.6 nm with IgA (Fig. 4A through C). Therefore, IgM and IgA affected EV size, whereas IgG did not.

The number of EVs produced per cell was analyzed based on the concentration of cells in 30 mL of PBS after recovery from Sabouraud plates (see Material and methods), together with the particle concentration/mL after analyzing the samples by NTA. For cells cultured with IgM, IgG and IgA, there were fewer detectable EVs per cell than for the control (H99 in Sabouraud); this difference was significant for cells cultured with IgA, *P* value * < 0.05, Fig. 4D. H99 cultured with IgA produced ~0.39 EVs per cell, while cells cultured with IgG and IgM produced ~0.47–0.51 EVs/cell. H99 cultured without immunoglobulins produced ~0.94 EVs per cell.

### Gene expression profiles of H99 cultured with IgA, IgG, and IgM

As shown herein, the immunoglobulin isotypes had different effects on H99 Titan-like cell induction and EV production, albeit in different conditions. Since IgA affected H99 morphology and had a unique effect on EV size, we analyzed gene expression profiles of H99 cultured with each immunoglobulin isotype in Sabouraud and compared the results obtained with IgA to IgM and IgG.

#### IgA compared to control

**Fig 5:**
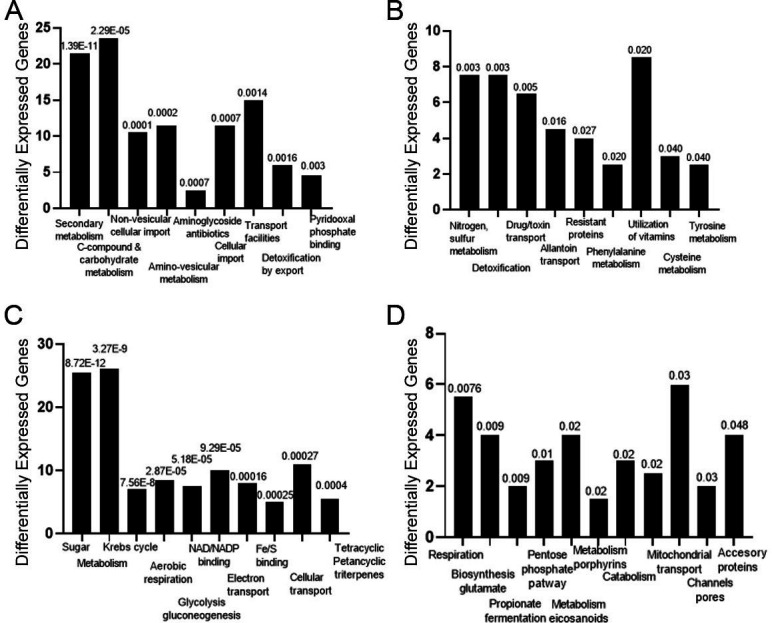
Gene expression changes in H99 cells cultured with human IgA and the control (Sabouraud) by RNA-seq. Gene ontology analysis of H99 gene categories and pathways overexpressed (**A and B**) and repressed (**C and D**) after culture with IgA compared with control (Sabouraud) after 6 h of incubation. Numbers above the histograms denote the *p*-value for differentially expressed genes, Fisher’s exact test with the significance level set to 0.05 and the Benjamini–Hochberg adjustment method.

After mapping, the reads and subsequent analysis of differentially expressed genes were analyzed using the DESeq2 algorithm. There were 292 overexpressed genes and 282 repressed genes in H99 cultured with IgA compared to the control (Fig. S1). Gene ontology analysis of the genes overexpressed after culture with IgA revealed genes involved in secondary metabolism, C-compound and carbohydrate metabolism, amino acid metabolism, and non-vesicular cellular import (Fig. 5A and B). More than 80 genes were related to metabolism, with CNAG_00735 (aldehyde dehydrogenase family seven member A1) being the most overexpressed. The protein encoded by this gene is a member of subfamily 7 in the aldehyde dehydrogenase gene family, which catalyzes the oxidation of aliphatic compounds and aromatic aldehydes. Other overexpressed genes included CNAG_03199 (FAD-dependent oxidoreductase), CNAG_00247 (alpha-aminodipic semialdehyde synthase), and genes involved in transport, including CNAG_03398 (solute carrier family 39 zinc transporter) and CNAG_06259 (MFS transporter, SP family, alpha glucoside: H symporter). These genes are reported in Table S1 (Supplementary material).

Among the categories of genes repressed after culture with IgA were sugar, glucoside, polyol and carboxylate anabolism, C-compound and carbohydrate metabolism, Fe/S binding, and electron transport (Fig. 5C and D). The repressed genes were also related to metabolism and the tricarboxylic acid pathway (citrate cycle, Krebs cycle, TCA cycle) as CNAG_02133, CNAG_06977, and CNAG_00797 (Fig. 5C and D). Other repressed genes were related to mitochondrial transport (cellular transport, transport facilitation, and transport routes), especially CNAG_03359 and CNAG_06407, and genes involved in channel/pore class transport and accessory proteins of electron transport and membrane-associated energy conservation (see Table S1). The classification by most representative categories of the overexpressed and repressed genes in the comparison of cells grown in the presence of IgA compared to the control is shown in Fig. S1.

#### IgA compared to IgG

**Fig 6:**
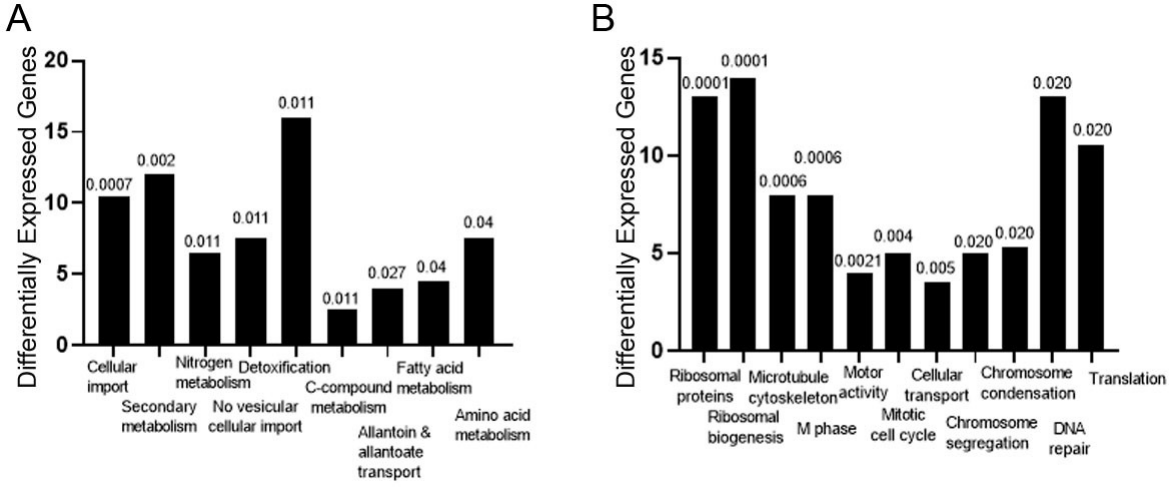
Gene expression changes in H99 cells cultured with human IgA and IgG by RNA-seq. Gene ontology analysis of H99 gene categories and pathways overexpressed (**A**) and repressed (**B**) after culture with IgA compared with cells cultured with human IgG after 6 h of incubation. Numbers above the histograms denote the *p*-value for differentially expressed genes, Fisher’s exact test with the significance level set to 0.05 and the Benjamini–Hochberg adjustment method.

We analyzed the H99 overexpressed and repressed genes after culture with IgA compared to cells cultured with IgG. After mapping the reads and subsequent analysis of differentially expressed genes using the DESeq2 algorithm, there were 360 overexpressed genes after culture with IgA compared to IgG and 426 repressed genes in the same conditions (Table S1; Fig. S2). Gene ontology analysis of genes overexpressed with IgG revealed genes involved in cellular import, secondary metabolism, nitrogen, sulfur and selenium metabolism, non-vesicular cellular import, and detoxification involving cytochrome P450. The three most overexpressed genes were CNAG_00749 (alternative sulfate transporter), CNAG_00541 (dimethylaniline monooxygenase), and CNAG_03340 (flavonol synthase). There were also four genes related to detoxification involving cytochrome P450: CNAG_01714, CNAG_03168, CNAG_03389, and CNAG_06249, which is involved in cell rescue, defense, and virulence (Fig. 6A).

Among the genes repressed by IgA was CNAG_04306 (vesicle transporter SFT2B). Repressed genes also included those involved in ribosome biogenesis, microtubule cytoskeleton, M phase, mitotic cell cycle, and tubulin-dependent transport (Fig. S2). IgG-induced genes were involved in transport, including CNAG_00749 (alternative sulfate transporter), CNAG_03339 (biotin transporter), and CNAG_01964 (OPT family small-oligopeptide transporter) (Fig. 6B).

#### IgA compared with IgM

**Fig 7:**
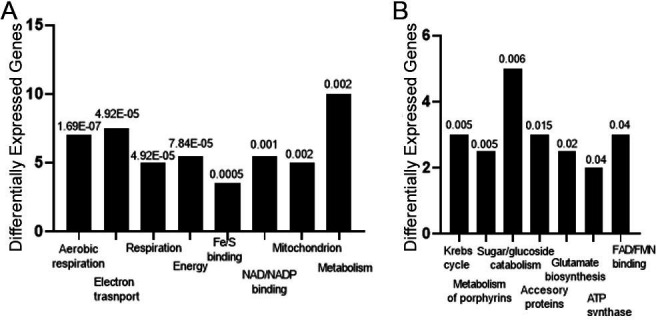
Gene expression changes in H99 cells cultured with human IgA and IgM by RNA-seq. Gene ontogeny analysis of H99 gene categories and pathways overexressed (**A and B**) after culture of IgA compared with cells cultured with human IgM after 6 h of incubation. Numbers above the histograms denote the *p*-value for differentially expressed genes, Fisher’s exact test with the significance level set to 0.05 and the Benjamini–Hochberg adjustment method.

We analyzed the gene expression of H99 after culture with IgA compared to cells cultured with IgM. After mapping the reads, subsequent analysis of differentially expressed genes was performed using the DESeq2 algorithm. There were 125 genes overexpressed after culture with IgM compared to IgA and three repressed genes in the same conditions (Fig. S3). The three genes repressed were CNAG_00030 (D-3-phosphoglycerate dehydrogenase), CNAG_00053 (hypothetical protein), and CNAG_00063 (histone H3). The genes overexpressed after culture with IgM compared to IgA were those involved in aerobic respiration, electron transport, respiration, Fe/S binding, NAD/NADP binding, and mitochondrion (Fig. 7A and B). The most overexpressed gene was CNAG_01742, aquaporin-associated protein (Table S1).

## DISCUSSION

The effect of human IgA on *C. neoformans* morphology has not been examined in detail previously, although one report suggested that its ability to inhibit Titan-like cell formation may be like that of IgM (28). The data presented herein show that after 24 h of culture with IgA, *C. neoformans* H99 cells were significantly smaller than those cultured with IgM, IgG, or PBS. There was no discernable effect of any of the immunoglobulin isotypes on H99 growth curves in the conditions used in this study. The effect of IgA on H99 resembled that which was previously observed with IgM (28), including that IgA inhibited Titan-like cell formation. Titan cells enhance virulence in the host (33, 34). Therefore, our data suggest that IgA, like IgM, may inhibit *C. neoformans* virulence characteristics associated with a Titan-like cell formation, perhaps via their ability to bind conserved carbohydrate and/or capsular determinants (37–40).

Numerous studies have revealed associations between perturbations in human serum immunoglobulin levels and cryptococcal disease status (26, 27, 41). In one study, levels of total IgG were higher in patients with cryptococcosis (cases) than controls, while levels of IgA and IgM were higher in controls than cases (42). Our data showing that IgG does not inhibit Titan-like cell formation, whereas IgM and IgA do, are suggest the hypothesis that IgM and IgA may enhance resistance to cryptococcosis. While smaller non-Titan-like cryptococcal cells might be more easily phagocytosed, cryptococcal seed cells, which exhibit size variability (43), appear to be more likely to disseminate.

More work is needed to understand the relationship between the effect of IgA on cryptococcal size, cell surface characteristics, and virulence *in vivo*. Nonetheless, a case–control clinical study showed that plasma IgA and laminarin–IgA levels were lower in individuals with, than without, HIV-associated cryptococcal meningitis (26). Therefore, our finding that normal human IgA can inhibit a Titan-like cell formation *in vitro* lends biological plausibility to the idea that it may have a host beneficial effect *in vivo*.

An important discovery in this study is that the immunoglobulin isotypes altered EV production. Previous work with human IgM (28) revealed distinct patterns of vesicle localization in H99 by electron microscopy; vesicles were observed within cryptococcal cells cultured with IgM and control, but not IgG, suggesting a connection between vesicle distribution and human IgM binding to H99. Although ultrastructure studies with IgA were beyond the scope of this study, our current results reveal a previously unknown factor, namely, that human immunoglobulin binding may affect *C. neoformans* EV biogenesis. Currently, we can only speculate on the explanation for this finding. If the immunoglobulins interact with EV lipids, membrane curvature and EV size could be altered, or if they interact with the cell wall, they could alter its permeability to vesicle passage and affect the number of detectable EVs. The gene expression changes induced by immunoglobulin binding could also impact pathways required for vesicle trafficking and/or biogenesis, although we do not have direct evidence for this. Nonetheless, we believe our finding that fungal EVs change in response to human immunoglobulins is novel and noteworthy.

To our knowledge, the effect of human IgA on fungal gene expression has not been explored previously. Human IgM, but not IgG, was previously shown to decrease expression of selected H99 genes that regulate stress response pathways and morphogenesis (28). Notably, a microarray-based transcriptional analysis found that protective and non-protective GXM-binding mouse monoclonal antibodies differentially altered *C. neoformans* gene expression and modulated fungal metabolism (44), and a mouse pneumococcal capsular polysaccharide monoclonal antibody altered pneumococcal quorum sensing *in vitro* and expression of genes required for adaptation and resistance to stress *in vivo* (45, 46). In this study, we investigated the effect of normal human immunoglobulins on H99 gene expression. To focus on the effect of IgA, we compared the transcriptomes of H99 cultured with IgA to those of cells cultured with IgM, IgG, and without immunoglobulin (control). The experiment was performed in Sabouraud medium to enable us to isolate enough RNA. This would not have been possible with TCM, as it would have required large volume cultures at very low cell density and amounts of immunoglobulin that were not feasible.

When we compared cells grown with IgA to control (Sabouraud), the most overexpressed gene was aldehyde dehydrogenase superfamily ALDH7. ALDH7 proteins, which are highly conserved across species, catalyze oxidation of α-aminoadipic semialdehyde in lysine degradation, protect against hyperosmotic stress, and detoxify aldehydes in humans (47). Elevated expression of aldehyde dehydrogenase was observed as part of the metabolic adaptation to early murine cryptococcal pulmonary infection (48). Although the function of many of the genes identified in this analysis in *C. neoformans* is unknown, based on our findings, it is logical to posit that they may protect against toxic metabolites that arise during environmental adaptation and/or stress. Along these lines, IgA led to overexpression of CNAG_ 03199 (FAD-dependent oxidoreductase) and CNAG_02225 (glucan 1,3-β-glucosidase), along with more than 80 genes related to carbohydrate metabolism, amino acid metabolism, or secondary metabolism. Expression of a *Cryptococcus podzolicus* Y3 FAD-dependent oxidoreductase was downregulated by mycotoxin exposure (49), and CNAG_02225 (glucan 1–3-β-glucosidase) expression, along with that of other genes encoding extracellular proteins, was reduced in inositol pyrophosphate-deficient *C. neoformans* mutants (50). Other genes repressed by IgA were related to the Krebs cycle, mitochondrial transport, and energy conservation (e.g., CNAG_00061, CNAG_00797 and CNAG_01120 among others), as well as cellular and transport facilities, suggesting that IgA may affect H99 fitness by affecting mitochondrial function, EV transport, or glycolosis. Most notably, IgA repressed CNAG_002133 (6-phosphogluconolactonase), which is upregulated in *C. neoformans* Titan cell formation (20). Although this finding must be validated experimentally, it links IgA inhibition of Titan-like cell formation *in vitro* to gene expression. Collectively, in comparisons with control (Sabouraud), IgA-induced transcriptional changes are suggestive of a stress response in which virulence transformation and the ability to transition to greater energy utilization may be impaired.

Comparing the effects of IgA and IgG, the most overexpressed genes were related to secondary metabolism, followed by transport facilities and non-vesicular cellular import. These changes may be indicative of environmental adaptation or a stress response. In contrast, IgA-repressed genes were associated with M-phase and microtubule cytoskeleton and may be indicative of a defect in energy conservation and/or cellular stability. Notably, the most repressed gene, was related to vesicular transport, CNAG_04306, which likely encodes the vesicle transporter protein SFT2B. The biological role of SFT2B in fungi has not been defined, but in humans, this molecule is predicted to be involved in protein transport and located in extracellular exosomes (https://www.uniprot.org/uniprotkb/O95562/entry). Our data show that H99 cultured with each immunoglobulin isotype produced fewer EVs than the control (PBS), with IgA producing the fewest. Interestingly, EVs produced by cells cultured with IgA and IgM, which both inhibited Titan-like cell formation *in vitro*, were larger than those produced by IgG and the control. Exosomes are small EVs with diameters of 40–100 nm, whereas microvesicles are larger EVs in the 100–1000 nm range (32, 51–54). Although more work is required to understand these results and identify EV contents, our findings suggest that IgA may reduce EV production while forming larger vesicles, perhaps via repression of CNAG_04306. Other highly repressed genes in the IgA to IgG comparison were CNAG_03065 (ER-derived vesicles protein ERV14) and CNAG_01148 (FK506-binding protein 4). Deletion of *Erv14* in *Aspergillus* inhibited mycelial growth and extracellular protein secretion (55). FK506 inhibits calcineurin, which is required for *C. neoformans* virulence at mammalian temperatures (56).

Comparing the effects of IgA and IgM on H99, there were only 145 overexpressed and three repressed genes. Of the repressed genes, the most notable was CNAG_00063, histone H3, which was recently related to morphological changes in *C. neoformans* (57). The histone acetyltransferase Gcn5-mediated histone H3 plays a crucial role in completing the cryptococcal sexual cycle, yeast–hyphae morphogenesis, and subsequent sexual reproduction (57). Mutants with reduced histone H3 lysine 9 acetylation were defective in their response to stress conditions and sexual development and had reduced virulence (58). In addition, histone deacetylase mutants and/or inhibitors rendered *C. neoformans* more susceptible to antifungal drugs (59), suggesting that IgA-mediated effects might enhance the efficacy of antifungal agents as described for mouse monoclonal GXM antibodies (60). Although IgM and IgA had a similar effect on Titan-like cell formation, our data suggest that IgA may have a unique effect on *C. neoformans* virulence, perhaps via regulation of histone H3 and/or other deacetylases. The most overexpressed gene in cells cultured with IgA compared to IgM was CNAG_01742, an aquaporin-associated protein. *C. neoformans* A*QP1* has been implicated in metabolic homeostasis; its deletion led to accumulation of primary and secondary metabolites that were depleted when it was overexpressed (61). Therefore, like comparisons between IgA, IgG, and the control, compared to IgM, the IgA-induced transcriptional changes appear to reflect adaptation to stress.

Collectively, our RNAseq data revealed IgA-associated differences in gene expression compared to the control, IgM, and IgG. Notably, the IgA to control comparison revealed downregulation of a gene that was upregulated in Titan cell formation (CNAG_02133). Other differentially regulated genes mirror *C. neoformans* responses to stress and environmental adaptation. While we cannot link our findings to capsular, cell surface, or changes in virulence *in vivo* at this time, they are noteworthy and novel. We do not know how immunoglobulin induces gene expression changes, although like gene expression changes of mouse monoclonal capsular antibodies were associated with distinct *C. neoformans* binding patterns (44), they are most likely secondary to immunoglobulin cryptococcal cell surface and/or capsule binding (28). Comprehensive analyses of human immunoglobulin interaction with *C. neoformans* are needed to assemble a mechanistic explanation of our findings, but such work is beyond the scope of this study. We need to identify the *C. neoformans* specificity of the immunoglobulin isotypes, relate differential gene expression to morphological, phenotypic, biophysical, and/or changes in virulence, and determine the effect of the conditions used on our results. Therefore, we acknowledge that this report is observational and preliminary. Nonetheless, our results indicate that human IgA has a distinct effect on H99 morphology and gene expression *in vitro*, and that it differentially regulates genes that are involved in environmental adaptation, stress responses, growth, and energy utilization.

We believe our findings suggest a new paradigm for understanding the role of antibody immunity in cryptococcal virulence. The hypothesis that immunoglobulins may affect *C. neoformans* biology is plausible based on multiple studies showing perturbations in specific and natural immunoglobulin levels in patients with cryptococcal antigenemia and cryptococcosis. However, our study has important limitations. Since mucosal IgA is dimeric, and serum IgA is largely monomeric, more work is needed to determine the effects of monomeric and dimeric IgA on *C. neoformans* morphology and gene expression. Glycosylation of IgA may also affect its effect on *C. neoformans*. The RNAseq experiments were conducted at 30°^C^ and Titan-like cell-inducing experiments were performed at 37°C with different media. Environmental conditions, including media composition, temperature, pH, and other factors, affect Titan cell-like and vesicle formation (51) and are likely to affect gene expression. Therefore, we do not know if our results are applicable to other experimental conditions, and our future work will focus on conditions that more closely recapitulate human physiology. Despite these limitations, our data reveal new insights into human host*–C. neoformans* interaction and suggest that antibody responses may regulate gene expression in this important fungal pathogen. Overall, our findings indicate that host defense against *C. neoformans* is more complex than we have long assumed.

## SUPPLEMENTAL MATERIAL

Figure S1 spectrum.02008-24-s0001.tif The most significant enriched categories of overexpressed and repressed genes.

Figure S2 spectrum.02008-24-s0002.tif The most significant enriched categories of overexpressed and repressed genes.

Figure S3 spectrum.02008-24-s0003.tif The most significant enriched categories of overexpressed and repressed genes.

Figure S4 spectrum.02008-24-s0004.tif Cell body size.

Legends spectrum.02008-24-s0005.docx Supplemental figure legends.

Table S1 spectrum.02008-24-s0006.xlsx Genes that were significantly overexpressed or repressed in *C. neoformans* strain H99 after culture with human IgA, IgM, and IgG.

ASM does not own the copyrights to Supplemental Material that may be linked to, or accessed through, an article. The authors have granted ASM a non-exclusive, world-wide license to publish the Supplemental Material files. Please contact the corresponding author directly for reuse.
