# Supplementary figures and images for: Effects of human immunoglobulin A on Cryptococcus neoformans morphology and gene expression
Source: Microbiol Spectr. 2025 Feb 21;13(4):e02008-24. doi: 10.1128/spectrum.02008-24 (PMC11960444; doi:10.1128/spectrum.02008-24)

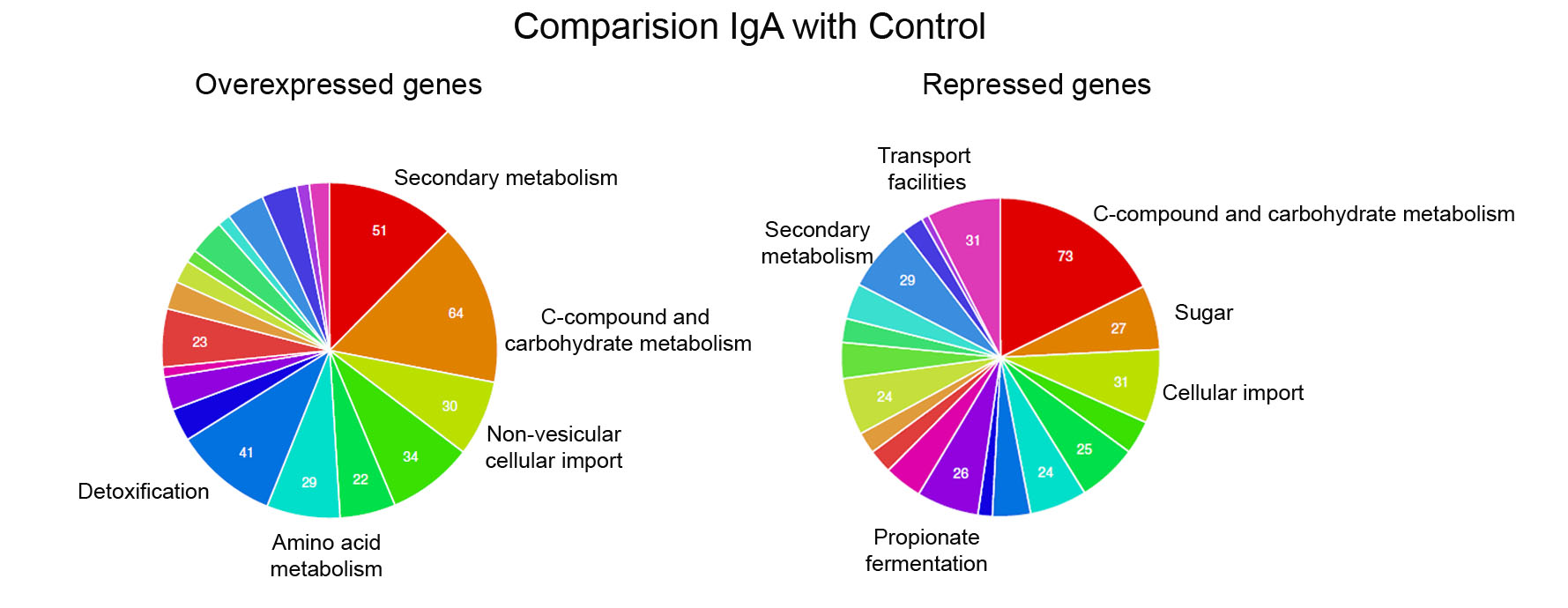

Supplement: Figure S1 — The most significant enriched categories of overexpressed and repressed genes. [file spectrum.02008-24-s0001.tif]

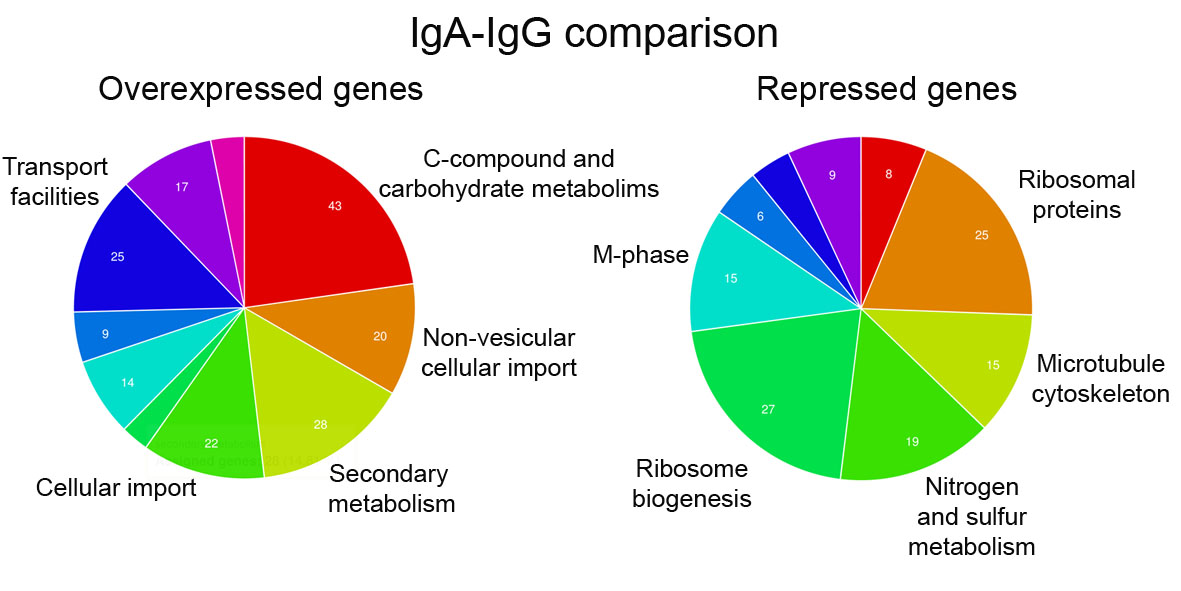

Supplement: Figure S2 — The most significant enriched categories of overexpressed and repressed genes. [file spectrum.02008-24-s0002.tif]

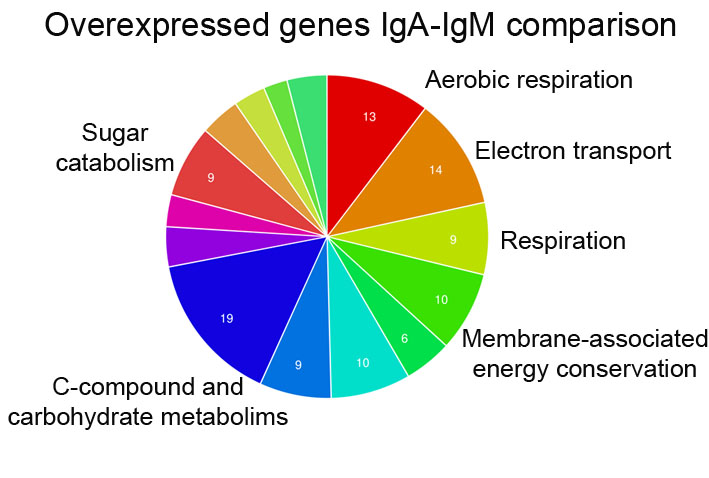

Supplement: Figure S3 — The most significant enriched categories of overexpressed and repressed genes. [file spectrum.02008-24-s0003.tif]

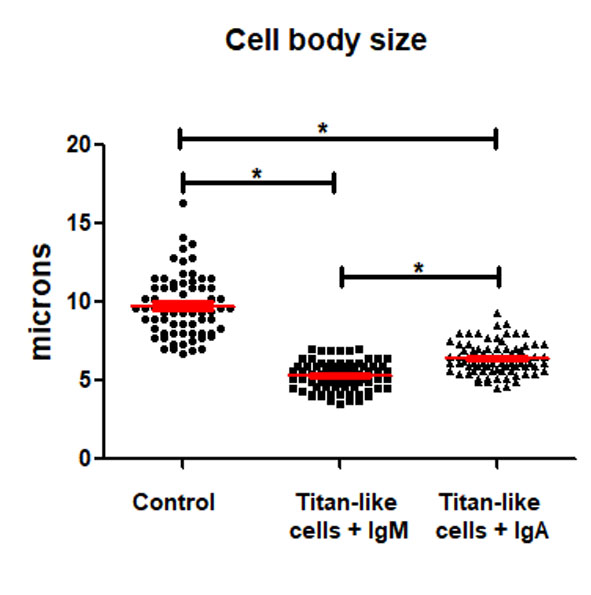

Supplement: Figure S4 — Cell body size. [file spectrum.02008-24-s0004.tif]
