# Supplementary material for: Effects of human immunoglobulin A on Cryptococcus neoformans morphology and gene expression
Source: Microbiol Spectr. 2025 Feb 21;13(4):e02008-24. doi: 10.1128/spectrum.02008-24 (PMC11960444; doi:10.1128/spectrum.02008-24)
Supplement: Legends — Supplemental figure legends. [file spectrum.02008-24-s0005.docx]

**Supplementary material**

**Table S1.** Genes that were significantly overexpressed or repressed (fold>2 with P <0.05) in *C. neoformans* strain H99 after culture with human IgA, IgM and IgG.

**Figure S1.** Representation of the most significant enriched categories of overexpressed and repressed genes comparing *C. neoformans* cultured with IgA and control.

**Figure S2.** Representation of the most significant enriched categories of overexpressed and repressed genes comparing *C. neoformans* cultured with IgA with IgG.

**Figure S3.** Representation of the most significant enriched categories of overexpressed and repressed genes comparing *C. neoformans* cultured with IgA with IgM.

**Figure S4.** Cell body sizes of *C. neoformans* grown in TCM medium in presence of IgA and IgM. The images were obtained on a Leica microscope with the 40X objective. Asterisks indicate significant differences (* p<0.05). One-way ANOVA and Dunnett´s multiple comparisons test. The bars represent the standard errors of the means. The experiments were performed three different times on three different days.
